# On Regimen and Longevity: Comprising Materia Alimentaria, National Dietetic Usages, and the Influence of Civilization on Health and the Duration of Life

**Published:** 1844-07-01

**Authors:** 


### On Regimen and Longevity: comprising Materia Alimentaria, National Dietetic Usages, and the Influence of Civilization on Health and the Duration of Life. By *John Bell*, M.D.

The object of this little work is stated to be, to set forth the conditions and attainable means by which communities are kept in health, and longevity attained, rather than to afford minute specifications of the arts by which a luxurious and pampered individual may compromise between his appetites and his health, and procure enjoyment, if it were possible, without complying with the laws through which alone it is procurable.

Accordingly, a considerable portion of the book is devoted to the consideration of the dietetic usages of ancient and modern nations, and of the nature of the substance used for food in most parts of the inhabited globe. It is to this part of the subject, a very interesting one, that we shall principally confine the present notice.

With regard to the connection between health and longevity, and personal enjoyment and prolonged usefulness, the subjects of which the author first treats, we need say nothing. It requires no ghost to tell us, that without health there can be but little personal enjoyment, and without long life there can scarcely be *prolonged* usefulness.

*Influence of Civilization on the Duration of Life.*—In proportion as civilization advances it would appear that the mortality decreases. This would, at first sight perhaps, not seem so obvious to those who consider the deleterious effects upon the health produced by the constant strife for the means of existence rendered necessary by the increased density of the population. But, in proportion as civilization advances and the relations of men to each other become multiplied and complex, a more methodical and continued attention is paid to all suitable measures, calculated to prevent the injurious operation on the public health of personal or local impurity and taint. The rich inhabitants of spacious mansions are obliged, for their own sakes, to attend to the condition of the dwellings of their pauper neighbours. At any rate, whatever may be the reasoning, such, if we are to put any trust in statistical tables, is the fact.

In the city of Geneva, where an accurate account has been kept of the population, births, and deaths, for nearly the three last centuries, according to M. Mallet, the mean duration of the lives of the citizens of that republic, from 1560 to 1600, was twenty-one years and two months. In the seventeenth century, or from 1600 to 1700, the mean life was twenty-five years, nine months; and during the period from 1701 to 1760, it had increased to thirty-two years, nine months. This estimate of M. Mallett, strongly as it marks the difference in the duration of human life, in the above periods, is below that of some preceding writers, who did not make allowances for certain defects and omissions, which he points out, in the official tables. In 1833, the mean duration of life for the people of Geneva was forty years and five months; being nearly twice as long as it was rather more than two centuries before.

We have no means of comparing the mean duration of life in modern with that of ancient times, but according to Dr. Southwood Smith, whose opinion is founded on a document recorded by Domitius Ulpianus, Secretary to the Emperor Alexander Severus, the probable duration of life in Rome among the better classes was thirty years. Now the mean duration of life for the whole of the people of Great Britain is about forty-five years, which would give them an advantage over the Roman citizens in the time of Ulpianus, or three hundred years after Christ, of fifteen years. In France the mean duration of life in the classes more comfortably situated, is forty-two years, or twelve more than the people of Rome in corresponding circumstances.

According to M. Villermé, the mortality in Paris during the fourteenth century was one in sixteen ; at present the mortality, even in the poorest districts, is stated to be one in twenty-four. In Russia, many parts of which remain in a state of semi-barbarism, the mortality is one in 27 ; whilst in great Britain, where civilization has attained to a very great height, it is only about one in 44.

Dr. Bell carries the principle still further, and takes the effects produced by war :—

"In war between nations, the people are thrown back towards barbarism, by the ascendancy of the impulses of destructive courage over the better sentiments; and if the intellect manifests activity, it is chiefly under these impulses. The entire state of things is antagonist to civilization and to religion: it is eminently so to health and life." Besides the loss of life in the field of battle, and that caused by fatigue, exposure and privations, the country is deprived of its labourers, the fields are uncultivated, the amount of the means of subsistence lessened. The effects of a long-continued war are shewn even in the next generation, as was the case with the French youth drafted for the army after the general peace. In 1826, out of 1,033,422 young men drafted to serve in the army, 380,213 were sent back, because they fell short of even the diminutive stature of four feet ten inches French.

*National Dietetic Usages.*—In opposition to the usual opinion, Dr. Bell maintains that those who eat much flesh meat and in greater proportion than vegetable food, are less civilized in every sense of the word, than those who derive the greater part or all of the aliment from their vegetable kingdom. "A broader contrast," he says, "can hardly be furnished, in this respect, than between the Esquimaux and the Laplander—the first, eaters of seal and walrus, and the second, of reindeer—and the Hindoos and Chinese, so many millions of whom live exclusively on rice." But if we compare the rice-eating Hindoo with the Englishman who rejoices in the consumption of beef and mutton, we should hardly award to the former the superiority in civilization. The author, however, who evidently has no great relish for animal food, goes on to assert that the greater number of people in all ages have used, and continue to use, vegetable aliment alone ; if any meat is taken it is not a daily allowance, and its proportion is small.

The ancient Greeks subsisted mainly on vegetable food, though animal food was undoubtedly used by them at least occasionally, as Homer so often specifies the kinds of meat served up at the repasts of his heroes, as when Agamemnon, at an entertainment which he gave to Ajax, presented this latter with the chine of an ox, as a reward for his valour : and Alcinous in a banquet fed his guest upon beef. But the very emphatic mention of these things is considered to mark their rarity, and to show that they were only served up to those in high rank and power. It has been observed, as illustrative of the daily fare of those whom the heads of the house were not particularly desirous of pleasing,—that the suitors of Penelope, though given to all sorts of pleasure, are never entertained with either fish or fowl, or any delicacies.

The diet of the Romans in the early ages of the republic was extremely simple, consisting mainly of vegetable aliment of the commonest kind, such as pulse or barley. Afterwards, however, the latter was replaced by wheat, and barley was only used in cases of necessity, from the failure of the wheat crops, or as a punishment to the soldiers who had misbehaved. Thus, we learn, that in the second Punic war, the cohorts which lost their standards had an allowance of barley assigned to them by Marcellus. Augustus Cæsar commonly punished the cohorts which gave ground to the enemy, by a decimation, and allowing them no provision but barley.

As Rome increased in wealth and power, however, the diet of the citizens underwent a great change; not only was animal food freely taken, but the Romans indulged in a much greater variety than is acceptable to our modern notions. Fricasseed sucking puppies were in great request, and water-rats, snails, and maggots fattened on old timber, were among their greatest dainties. But most of our readers are without doubt too well acquainted with the description of the supper in the style of the ancients, in Peregrine Pickle, to render any further account necessary.

In the East, the food is generally vegetable, rice, perhaps being the article in greatest use.

In Upper Egypt, where they cannot procure rice, they make a hasty meal on boiled horse-beans, or of lentils steeped in oil. Onions are used to an incredible extent. Dates supply them with sustenance part of the year; and in Summer, the vast quantities of gourds and melons which are then produced, place within their reach an agreeable variety. Their drink is the water of the Nile, more or less purified, with the occasional addition or alternation of buffalo milk.

In Abyssinia, however, the people evince a marked *penchant*, not only for flesh meat, but for raw flesh cut out of an animal alive, and while the fibres are yet quivering.

"The favourite portion is called the *shulada*, and is cut out, on each side, from the buttocks, near the tail. As soon as these are taken away, the wounds are sewed up by these surgical butchers, and plastered over with cow dung. The animal, which had been thrown down before, and during the operation, is now allowed to rise, and is driven forward on its journey. The fashionable parties at Gondar, the capital of Abyssinia, are served with *brinde*, or raw meat, with the same hospitable feeling as, in our part of the world, they would be with venison chops, done just to the turn. The animal, a cow or a bullock, is brought to the door, and the dainty pieces cut off in the manner above described. But, on this occasion, the animal is killed, before doing which, all the flesh is cut off in solid square pieces, without bones or much effusion of blood. Two or three servants are then employed, who, as fast as they can procure the *brinde*, lay it upon cakes of teff placed like dishes down the table, without cloth or anything else beneath them." 60.

The Arabs chiefly subsist on the milk and butter obtained from their flocks and herds, together with a scanty supply of grain converted into flour. Camel's flesh is rarely eaten; a kid or lamb is often prepared out of compliment to a distinguished guest, but a person of less note is treated with coffee, or bread and melted butter.

In India and the greater part of China, rice is the staff of life; the Chinese, however, allow themselves a wide range in animal food, not so much perhaps on account of their natural propensities, as on account of the scanty supply of ordinary flesh meat and still more of food of any kind in so redundant a population as that of China. This will serve to explain their eating dogs, rats, and almost every kind of animal flesh. A favourite luxury with the rich consists of soups made with the gelatinous substances, sea-slug, bird's nests, &c. imported from the islands in the China and Java seas.

In strong contrast with the almost exclusively vegetable diet of the Southern Asiatics, of whom we have hitherto spoken, is the large, if not exclusive, use of animal food by the inhabitants of Northern Asia—Tartary and Siberia. The favourite food of the Tartars is horse-flesh; the horses being carefully fattened up for the tables of the rich. As, however, the number of horses is limited, and the flesh is consequently expensive, the poorer classes, and the wandering tribes in general, must put up with mutton in its stead.

In Siberia, especially the northern parts, the employment of the people is hunting and fishing, chiefly for the sake of food, partly to procure furs for the purposes of clothing and trade. They live chiefly on soured cow's milk, mare's milk, and horse flesh. Bread is unknown among them. Fat is the greatest delicacy; and they eat it in every possible shape, raw and melted, fresh and spoiled. The inner bark of the larch, and sometimes of the fir, is grated and mixed with fish, a little meat, and milk, or fat in preference, and made into soup. They are also excessively fond of tobacco, which is used both by men and women, who swallow the smoke and so bring on a species of stupefaction.

In northern and central continental Europe, the mass of the population, that is the poorer classes, subsist in great measure on vegetable food, and that of the second or inferior of the cereal grains, viz.—rye, seasoned with the products of the dairy, and a small portion of meat or fish. In southern Europe maize, to a certain extent, takes the place of rye, and wheat is more freely used than it is in the North.

England boasts of the large proportion, comparatively to other countries, of animal food consumed by her inhabitants. It is estimated that wheat is supplied at the rate of a quarter, or eight bushels, for every individual in the kingdom. The proportionate quantity of flesh meat has not been ascertained; but, according to McCulloch, it is said to be for the people of London 107 pounds per individual throughout the year. This statement, however, we fear does not give a very correct view of the state of the operatives in large towns, or of the labourers in many agricultural districts, as in Essex or Sussex. In Ireland it is well known that the potato forms the chief subsistence of the abounding population. It is the hard fate of the Irish people, or the great majority of them, to be tantalized with an abundance on their own fields, of live stock, which they cannot themselves convert into food, but must sell to meet other requirements. Even the fatted pig, so often the companion of the children in the poor man's cabin, is in due time taken to market and sold, to be killed and salted for exportation. "It has been estimated, that the entire amount of imports of alimentary substances, vegetable and animal, into Great Britain from Ireland, in one year has been as high as ten millions of pounds sterling."

In the United States of America, according to Dr. Bell, the alimentary products are most abundant, and their consumption placed within the means of nearly all classes. Even the slave population of the South is better fed than the peasantry of any part of continental Europe, and luxuriously compared with a large proportion of the operatives in Great Britain. A full supply of animal food, usually bacon or salt pork and salt fish, with corn bread, is allowed to the slave; to which is added, either the Irish, or still more commonly farther South, the sweet potato; and, instead of corn, rice in the lower districts of Carolina and Georgia. In Virginia and the West, fresh meat is given to them not unfrequently. To most of them is allotted a piece of ground (a patch) for a garden, in which they grow sundry vegetables and fruits for their own use, and not seldom for that of their masters, by whom they are paid at a fair price. Poultry and eggs, which they also have of their own, are more generally sold by them, either to their master's family or at the nearest village or court-house; and with the money they purchase groceries and other minor luxuries, or articles of personal adornment. The fruit, which they raise in the largest quantities for their own consumption and for sale, is the water-melon. The house slaves partake of the fare of their superiors, with the exception of a more restricted use of wheat bread; but this cannot be called a privation among a people with whom, as in the case of those of the South and West, maize is the bread-corn, and the preferred one of the country.

If this be the diet of the slaves, that of the free white population ought to be most luxurious. In fact the author owns " that the people of the United States are in a large majority of them, overfed." The artisan in the city, and even the hired labourer in the country, eats meat oftener in the day than many of the farmers, owners of the land, in France, and substantial renters on the shores in Italy, eat in the week. Dr. Bell estimates that for every pound of beef or veal consumed in Great Britain, there are nearly three pounds consumed in the United States.

Having so much to eat, of course such a " go-ahead " nation considers it necessary not to waste too much time in masticating it; the following description of a Yankee meal being by an American, cannot be considered as exaggerated or prejudiced.

" With such a superabundance, as I have already said, of aliment of all kinds, procurable by all classes above destitution, it is natural that the Americans should be great eaters; one man consuming as much animal food in a day as would support three labouring men in Europe; and, together with vegetables and bread, taking also his glass of milk and no small quantity of pie or pudding, with often fruit afterwards. A man in harvest time, in almost any of the States, eats at his three meals, more, in nutritive amount, than would constitute luxurious living for eight East Indian or Chinese palanquin bearers for a week. In addition to the quantity, the time for consumption of food by our people is surprising, the latter being, however, in its brevity, in the inverse ratio of the former. Often, also, the rapidity with which a meal is dispatched seems but a signal for entire cessation from all labour, even that of thought, for some time afterwards. Thus, it is common enough for men of active business habits to make an onslaught on a well furnished table for about five to ten minutes, during which brief period they swallow, we will not say masticate, for they seem to consider their teeth as quite unnecessary instruments, with a fearful rapidity, parts of half a dozen of dishes. This feat accomplished, for really a simple Hindoo or Chinese would suppose it must be a piece of jugglery, these thankless consumers of the gifts of Providence, in place of rushing out from the table to their several marts of trade, as their first inordinate haste would seem to indicate, will be seen to seat themselves very leisurely, and, with their feet up and head thrown back, to puff away at their segars, for the next hour, with a gravity and an appearance of want of all care, which would do credit to the most orthodox follower of Mohamed, when enjoying his modicum of opium, and perchance dreaming the while of his being suddenly made a pasha of three tails, and having the plunder of a province at his disposal. But not to smoking only or the more noxious in itself, and more obnoxious to others, chewing of tobacco, do our people rely for helping digestion, as they call it, and for rousing their dormant sensibilities after their anaconda repast." 96.

The next portion of the work is devoted to the consideration of the various articles of vegetable and animal food: with these we have no occasion to meddle, as our readers will meet with fuller and more elaborate accounts of them in the works of Pereira and others; we may however make a remark or two on the subject of the potato.

The Potato (Solanum Tuberosum) is indigenous to Chili and Peru, in which countries it grows wild; the flowers of the wild plant, however, are said to be always pure white, free from the purple tint so common in the cultivated varieties. For some time after their introduction into England, potatoes were looked upon as a great delicacy, and cultivated by a very few. The Royal Society, in 1663, encouraged a more extensive cultivation of them, as a means of preventing famine. Previously, however, to 1684, they were raised only in the gardens of the nobility and gentry; but in that year, they were planted, for the first time, in the open fields in Lancashire—a county in which they have ever since been very extensively cultivated. Their growth was more rapidly extended in Ireland than in England, and they have long furnished from two-thirds to four-fifths of the entire food of the people of Ireland. Potatoes were not raised in Scotland, except in gardens, till 1728.

Some of the good people of Scotland were at first opposed to the new vegetable on the grounds that "potatoes are not mentioned in the Bible." In like manner, some of the priests in the Ionian Islands, at a later period, manifested their hostility, by alleging that the potato was the forbidden fruit, the cause of man's fall; and of course its use was both immoral and irreligious.

In France much of the success of the potato was due to the exertions of Parmentier, who persevered amidst open opposition and ridicule of all kinds. For a while, the king, Louis XVI, and his court wore the flower of the potato in the button-holes of their coats, as a means of enlisting popular favour on its side.

In Switzerland this vegetable is a leading article of food amongst the peasantry; in Poland it is cultivated to an extraordinary extent; in Italy, within the present century, its growth has been greatly encouraged. In the Tropics it does not commonly thrive unless it be grown at an elevation of 3,000 or 4,000 feet above the level of the sea, so that it can never come into very general use in those regions. In the United States, potatoes form a regular part of the daily food of a vast majority of the inhabitants.

It has been well remarked by Mr. McCulloch: "So rapid an extension of the taste for, and the cultivation of an exotic, has no parallel in the history of industry; it has had, and will continue to have, the most powerful influence on the condition of mankind."

With regard to the proportionate nutritive properties of potatoes and wheat, and the amount per acre of each produced, deductions have been made as to the number of persons that could be supported on an acre of land planted with the former, compared with those that might be supported on an acre sown with wheat.—By some, the proportion is stated to be as high as six to one, and by others as only two to one. M'Culloch supposes four pounds of potatoes to be equal to one pound of wheat, now the average produce of potatoes in Ireland is stated to be 82 barrels, or 22,960 pounds the Irish acre, and if we divide this by four, we have the standard in nutritive power of wheat, as 5,740 pounds. The average produce of wheat by the Irish acre, is estimated by Mr. Young at 4 quarters, or about 1,920 pounds, or only about one-third part of the solid nutriment afforded by an acre of potatoes. Even in Great Britain, the soil of which is better adapted to the growth of wheat, and thought to be less suitable to that of the potato, it admits of demonstration that "an acre of potatoes will feed double the number of individuals that can be fed from an acre of wheat."

It is clear, therefore, says Mr. McCulloch, that the population of a potato-feeding country may become, *other things being about equal,* from two to three times as dense as it would have been had the inhabitants fed wholly on corn. But it is exceedingly doubtful whether an increase of population, brought about by a substitution of potato for wheat, is desirable. Its use as a subordinate or subsidiary species of food, is attended, continues Mr. McCulloch, with the best effects—producing both an increase of comfort and security; but there are certain circumstances inseparable from it, which would seem to oppose the most formidable obstacles to its advantageous use as a prime article of subsistence. Potatoes are a crop that cannot be kept on hand; they are irregular in the amount of produce, more so than wheat; they are not capable, owing to their bulk and weight, and difficulty of preserving them on ship-board, of being imported in quantities when the home supply is deficient, or of exporting them when in excess.

Various estimates have been made of the entire value of potatoes annually consumed in Great Britain and Ireland. According to McCulloch, the yearly produce of potatoes may be taken at *twelve millions* sterling. In France it has been estimated that the average annual product, in three years, of potatoes and chesnuts, was nearly one hundred and thirty millions of bushels.

The final Chapter of the work is devoted to the consideration of longevity; from this we can only extract a short passage respecting the relative mortality of different nations, merely remarking that the estimates are not to be taken as, strictly speaking, accurate.

"The absolute mortality of the Russians, was, within the last twenty years 1 in 27; the Prussians 1 in 36·2; the French 1 in 39·7; the Dutch 1 in 38; the Belgians 1 in 43·1; the English 1 in 43·7; the Sicilians 1 in 32; the Greeks 1 in 30. We have no estimate of the mortality of the people of the United States. In Philadelphia, the annual mortality is 1 in 42·3; in Boston, 1 in 45; in New York, 1 in 37·83." 419.

The work, without much pretention to originality, is interesting and amusing.

